# Spatial Uniformity Assessment of Particles Distributed in a Spherical Fuel Element Using a Non-Destructive Approach

**DOI:** 10.1038/s41598-019-44383-0

**Published:** 2019-05-27

**Authors:** Xuzhi Zhao, Yahui Peng, Xian-gang Wang, Libing Zhu, Houjin Chen

**Affiliations:** 1 School of Electronic and Information Engineering, Beijing Jiaotong University, Beijing, China; 2 Institute of Nuclear and New Energy Technology, Tsinghua University, Beijing, China

**Keywords:** Nuclear fuel, Electrical and electronic engineering

## Abstract

In many manufacturing procedures, a large number of identical particles need to be disseminated uniformly into a given space. The uniformity of the spatial distribution of the particles can be critical to the properties of the final products. We proposed an image processing-based non-destructive technique to evaluate the particles’ spatial uniformity in a spherical space imaged with computed tomography. Both graphic (qualitative) and numerical (quantitative) methods were developed to demonstrate the (non-) uniformity of the particles. Simulation results indicated that the technique helped detecting the non-uniformity in the particles’ spatial distribution accurately. We conclude that the proposed technique can be used to test whether a number of particles in a sphere are uniformly distributed statistically and graphically.

## Introduction

In many manufacturing procedures, it is often that a large number of small and identical particles are disseminated into a given space^1–3^. Usually, the spatial uniformity of the particles is critical since the properties of the final products can be substantially influenced by the distributive patterns of the particles^4,5^. For example, a uniform distribution of nanoparticle reinforcement in metal matrix is a crucial factor to better mechanical properties of metal matrix nanocomposites^6^. Therefore, how to evaluate accurately the spatial uniformity or homogeneity of particles distributed in a media is an important issue for quality control and for improvement of the manufacturing technologies.

In manufacturing processes, however, whether the particles are distributed uniformly in a space is often subjectively assessed by human observers^4,6^. Such methods can be time-consuming, tedious, and most importantly, prone to human errors^7^. Quantitative and reliable techniques are needed to evaluate whether the distribution is uniform to achieve better quality control and/or to improve the manufacturing technologies of the products.

Previous studies assessing the degree of spatial uniformity of particle distributions can be found in the material, ecological, environmental, and astronomy science studies^8–12^. However, in most of the studies, particles were presumably distributed in a simplified 2-dimensional rectangular area, and the uniformity indices proposed were often application-specific^6,13^. Existing techniques include image metrics based on local density and its statistics, distance-based indices, and others^8^. For particles disseminated in a sphere, Voronoi tessellation and Delaunay triangulation have been used to assess the spatial uniformity^14,15^. However, that method could not pinpoint the locations of non-uniform particles clusters.

**Figure 1:**
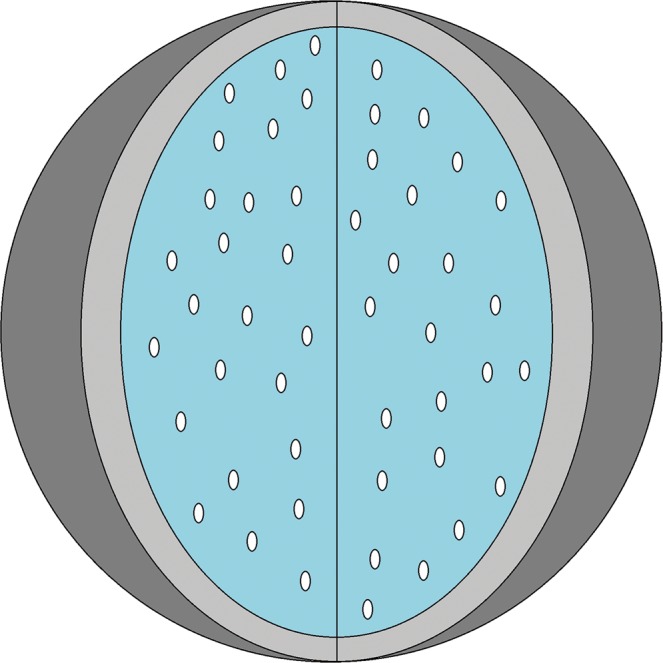
An illustrative sketch of a SFE containing a large number of TRISO-coated particles.

We propose a new technique to evaluate the spatial uniformity of a large number of identical particles disseminated in a three-dimensional spherical space quantitatively. The problem comes from the fabrication of spherical fuel elements (SFEs) for high-temperature gas-cooled nuclear reactors (HTGRs)^16,17^. Up to about 12,000 tri-structural isotropic (TRISO)-coated particles, 0.92 mm in diameter, may be disseminated into the fuel zone of 50 mm in diameter, and surrounded by a 5 mm-thick fuel-free layer of carbon as the supporting media (Fig. 1). The uniformity of the particles is critical since it may influence the thermodynamic and mechanical properties of the fuel element. Previously, destructive methods were used to inspect the uniformity of the TRISO-coated particles in the SFE^18^. However, destructive methods often need to grind the SFE to its equatorial plane, a time-consuming and high-cost procedure, not to mention the radiation hazard that may pose unnecessary health risks to human and environment.

Comparing to previous studies, the main innovations of the current study include that a fully automated image-processing technique is used to detect and segment the TRISO-coated particles, that a formal statistical test using a rigorous algorithm is performed to determine whether the particles are distributed uniformly in a sphere, and that three plots can be generated to indicate the locations of the non-uniformity, if any, in the sphere graphically. The proposed technique is also non-destructive, which is important since destructive methods often involve the radiation exposure or other risks.

In the next session, we introduce how a SFE was imaged non-destructively. An image-processing method is then developed in Session 3 for accurate particle segmentation. With the locations of the particles, the uniformity of the particle distribution is assessed using the proposed method in Session 4. To validate the assessment method, a simulation study is designed in Session 5. After the results are presented in Session 6, we briefly discuss and conclude the study in the last session.

## Tomographic Imaging

**Figure 2:**
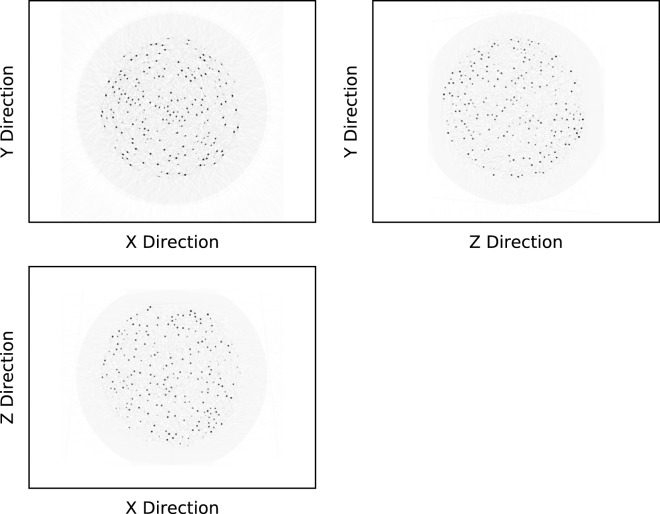
Reconstructed CT images of a spherical fuel element (SFE). The gray level is reversed for better presentation. The SFE is about 60 mm in diameter while the TRISO particles are 0.92 mm.

X-ray computed tomography (CT) was used to obtain the coordinates of the TRISO-coated particles. Due to the fineness of the large number (~12,000) of the particles, a CT system with high spatial resolution was employed. We used a cone beam CT machine with a flat panel detector to acquire data. And a paper cup was used to hold the SFE on the rotating stage of the CT to keep the SFE from moving during the CT scanning. We acquired 360 X-ray projections evenly with the projection interval of 1°. An isometric CT image was reconstructed with in-house software using the FDK algorithm^19^. The reconstructed image matrix was 1920 × 1920 × 1536. Since the diameter of the SFE was known, the magnification of the image could be determined. The voxel size of the reconstructed image was 73.6 μm. Three perpendicular planes of the reconstructed CT image of an SFE are shown in Fig. 2.

## Extraction of the Particles

**Figure 3:**
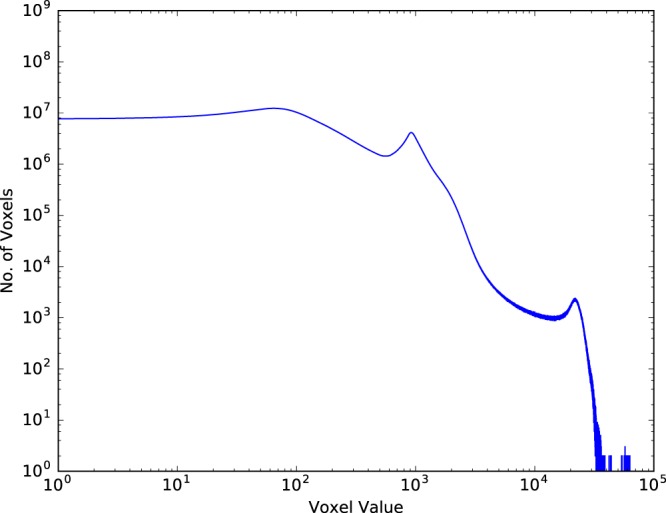
The number of voxels corresponding to different voxel values.

Figure 3 shows that the reconstructed CT image is consist of a large number of voxels with different voxel values. However, there is not an obvious threshold that can be used to separate the TRISO particles from the background. Therefore, we developed a two-step adaptive method to segment the TRISO-coated fuel particles automatedly.

In the first step, a global threshold determined with the Otsu method^20^ was used to select candidate voxels belonging to TRISO-coated fuel particles: only voxels with a value greater than the threshold were turned on. Because TRISO-coated fuel particles normally do not contact other particles, we then labeled the connected voxels to form volumes of interest (VOIs). The centroid of each connected VOI in the binary image was obtained as the center of TRISO-coated fuel particles.

**Figure 4:**
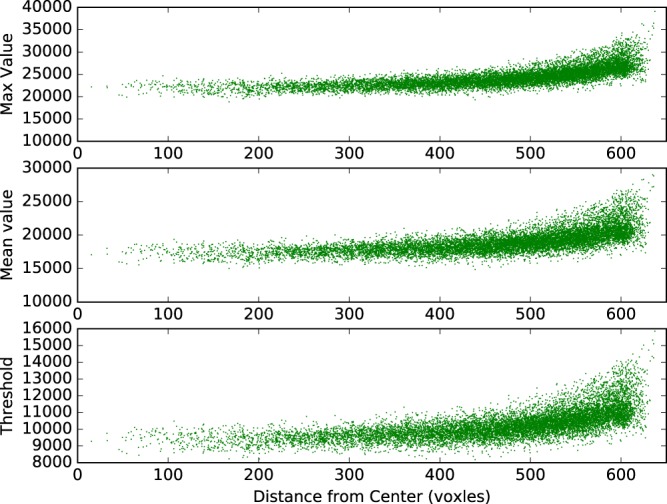
The maximum and mean voxel values of the volumes of interest obtained after the global thresholding and the adaptive thresholds for all the TRISO-coated fuel particles, as the location of the particles change from the center to the periphery of the SFE.

Due to the beam-hardening artifact in the CT image, the voxel values are often artificially reduced at the center of the image. In Fig. 4, the max voxel values and the mean voxel values rise as the distance between VOIs and the image center is increasing. To account for the beam-hardening effect, in the second step, the Otsu method was used again for each of the VOIs, and therefore, an adaptive threshold was generated for each VOI. In Fig. 4, the adaptive threshold is demonstrated that, as the distance from the image center is increasing, the threshold of the TRISO-coated fuel particles is also increasing, to compensate for the beam-hardening artifact.

## Uniformity Evaluation

We used the centroids of TRISO-coated particles to represent the locations of the particles and ignored the physical size in the following steps.

### The spherical coordinate system

**Figure 5:**
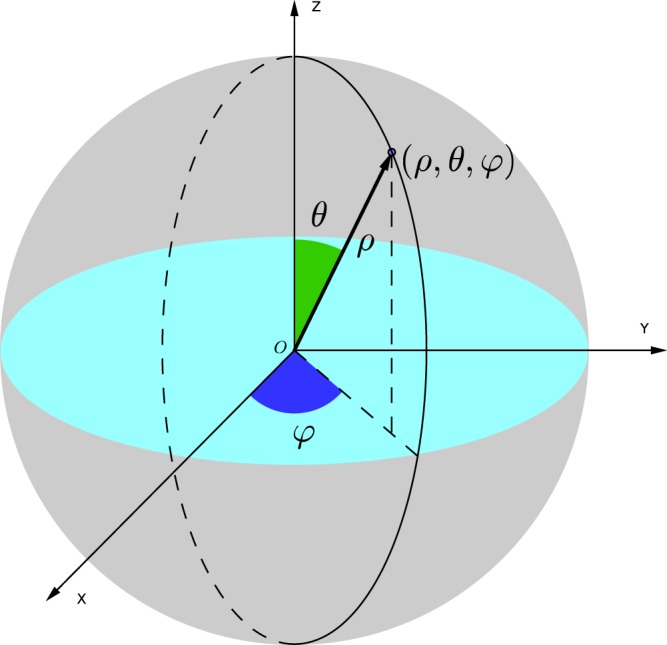
A spherical coordinate system to represent particle centroid in a spherical space.

To measure a large number of particles distributed in a spherical space, we decided to use a spherical coordinate system. Using the center of the SFE as the origin, we established a spherical coordinate system as shown in Fig. 5.

We used the radial distance *ρ*, the polar angle *θ*, and the azimuthal angle *φ* to determine a point in the spherical coordinates, denoted by the triplet (*ρ*, *θ*, *φ*). To represent a point uniquely using the triplet, we limited the range of the coordinates: 0 ≤ *ρ* ≤ *r*, 0 ≤ *θ* ≤ *π*, and 0 ≤ *φ* < 2*π*, where *r* is the radius of the sphere. We selected these ranges since all coordinates would have a positive value but other selections would not influence the following analyses significantly.

To standardize the parameters, we also normalized the radius of the sphere to unit. Therefore, readers should note that *r* = 1 hereafter, unless otherwise specified.

### Qualitative uniformity evaluation method

Projections of the uniformly distributed particles on the three coordinates in a sphere follow certain patterns. The radial distance *ρ* of uniformly distributed particles follows a power law in the range of [0, 1]. This is reasonable since the surface of the shell with a radius of *r* is proportional to *r*^2^. Similarly, the polar angle *θ* and the azimuthal angle *φ* of uniformly distributed particles follow the sin*θ* and uniform distributions, respectively. Therefore, to test whether the particles are disseminated uniformly in a spherical space, we calculated the histograms of the particle radial distance *ρ*, the polar angle *θ*, and the azimuthal angle *φ* and compared them with the power law, sine, and uniform distributions.

### Quantitative uniformity evaluation method

The qualitative method is subjective, i.e., different observers may give different conclusions even the same particle distribution is presented. Therefore, a more quantitative method is needed to evaluate the uniformity objectively.

The key trait of the uniformly distributed particles is that the histograms of the particles’ spherical coordinates follow the power law, sine, and uniform distributions, respectively, as described previously. Statistically, these hypotheses can be tested by the use of the Kolmogorov-Smirnov (KS) test for goodness of fit^21^. Therefore, we developed an algorithm that implemented the KS test for each of the three spherical coordinates.

Note that the null hypothesis is that the particles in the sphere are uniformly distributed. The null hypothesis should be rejected if any of the three KS tests yield a *p*-value smaller than a preset significance level *α*. Here we are making multiple comparisons. Because the three KS tests are independent, the Bonferroni correction^22^ can be used to adjust the critical value to compensate the multiple comparisons. For example, if we determine the desired significance level for the whole test is *α* = 0.05, then for each of the three individual KS test, according to Bonferroni correction, the significance level should be adjusted to *α*′ = 0.05/3 = 0.017.

## Simulation Studies

To validate the uniformity evaluation methods, we performed simulation studies. The first study assumed particles were disseminated in a spherical space uniformly, and we tested how often the quantitative uniformity evaluation method would mistakenly determine that the distribution was not uniform, i.e., the type I error rate. The second study assumed particles were disseminated in a sphere non-uniformly, and we tested how often the quantitative method would mistakenly determine that the distribution was uniform, i.e., the type II error rate.

To generate the desired uniformly and non-uniformly distributed particles in a sphere, we used a random number generator with rejection. For uniformly distributed particles, we generated three independent numbers from a uniform distribution between 0 and 1. The three numbers were then used as the coordinate triplet (*x*, *y*, *z*) in a Cartesian coordinate system. If the distance between the points (*x*, *y*, *z*) and the center (0.5, 0.5, 0.5) was smaller than or equal to 0.5, then the coordinates would be kept, otherwise, rejected. For non-uniformly distributed particles, numbers would be drawn from non-uniform distributions. In this study, we used normal distributions with the mean of 0.6 and the standard deviation of 0.5.

Seven different numbers of particles were generated in the simulation studies: 100, 200, 500, 1000, 1500, 2000, and 2500. For each of them, 1000 trials were simulated to calculate the error rates. We then repeated the studies 25 times to obtain the mean and standard deviation of the type I and type II error rates.

## Results

**Figure 6:**
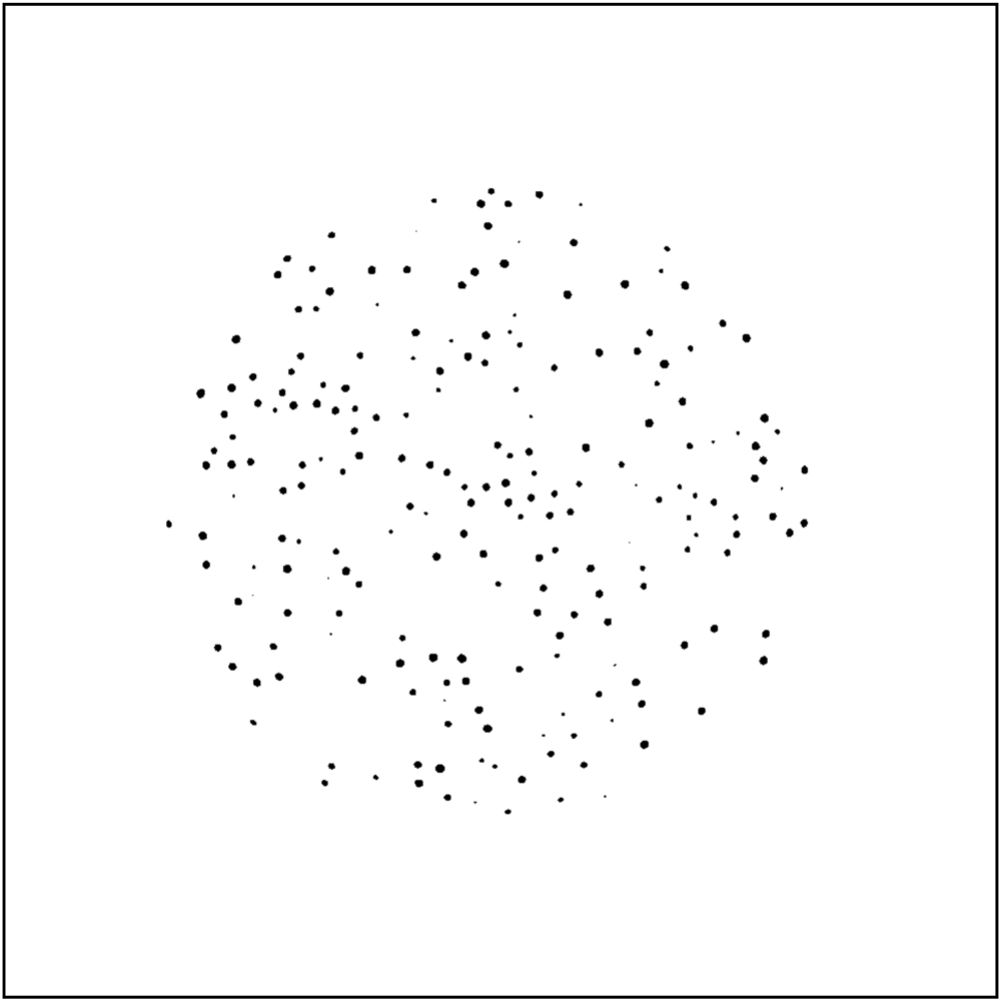
The segmented particles at the plane of z = 0. For better visualization, gray level is reversed.

A 2-dimensional slice of the SFE with the segmented TRISO-coated particles is shown in Fig. 6. By labeling connected regions in the SFE, we obtained the number of particles that was 11,603. The relative error to the nominal number (12,000) was 3%, which was within the manufacturing error limit.

**Figure 7:**
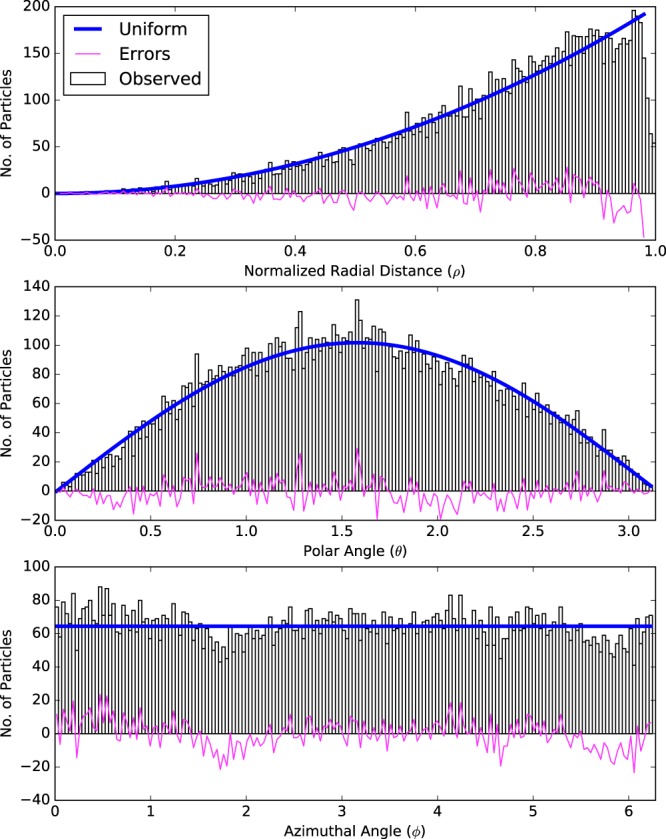
Coordinate histograms of the centroids of the TRISO-coated fuel particles in the SFE. The thick solid curves are the theoretical curves for uniformly distributed particles. And thin curves are the difference between the histograms and the theoretical curves.

Coordinate histograms of the TRISO-coated fuel particles are shown in Fig. 7. The corresponding theoretical curves for uniformly distributed particles are shown on top of the histograms for visual comparison. The differences between the histograms and theoretical curves are highlighted, as well. Since the outmost particles may not form a complete shell, we excluded the outmost 10% particles in the distribution of the radial distance.

The quantitative uniformity evaluation method indicated that the TRISO-coated particles was not uniformly distributed. Further statistical analysis revealed that the particle distribution was not uniform in the radial distance but uniform for the polar and azimuthal coordinates.

**Figure 8:**
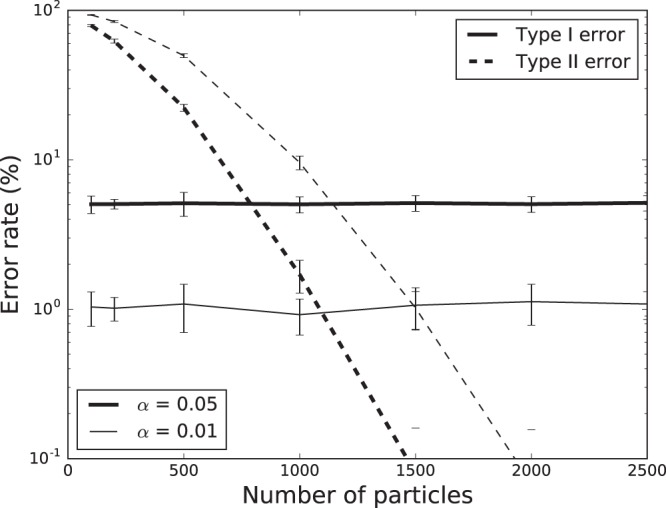
Type I and type II error rates for different numbers of particles in a sphere based on the simulation studies.

Figure 8 shows the type I and type II error rates for different numbers of particles in a sphere. The type I error rate appeared to be independent on the number of particles in the sphere. The level of the type I error rate was also consistent with the selected significance level *α*. However, the type II error rate demonstrated a strong trend indicating that the more particles, the less possible that the quantitative uniformity method would make type II errors. In other words, the power of the quantitative uniformity method increased with increasing number of particles in the sphere.

## Discussion

Previous studies on assessing particle uniformity in a given space often limited to 2-dimensional rectangular cases^6,13^. And commonly used uniformity indices were not applicable to general cases^6,8,13^. Besides, uniformity assessment of particles distribution in a sphere using Voronoi tessellation and Delaunay triangulation could not be used to indicate the location of non-uniform clusters^14,15^. We developed an image processing-based general technique to test whether particles disseminated in a 3-dimensional spherical space were uniformly distributed or not. A formal statistical analysis was developed to determine whether the distribution was uniform at a given statistical significance level, reaching a more convincing conclusion. In addition, a graphical method was presented to visually detect possible non-uniform patterns in a more intuitive way.

The simulation results validated the quantitative statistical method. The type I error rates were consistent with the significance level and did not change with the number of particles, while the type II error rates decreased with the increasing number of particles. These results indicate that quantitative statistical method is reliable and powerful in detecting non-uniformity in the spatial distributions of particles.

The non-uniformity of the TRISO-coated particles was caused by the distortion of the particle distribution along the radial distance. Close comparison of the histogram and theoretical curve on the radial coordinate indicated that the particles were distributed more densely on the outer shells. Based on this observation, we tried to exclude outer particles in the SFE and found that only when 39% innermost particles were included in the analysis, was the distribution of particles uniform. This phenomenon probably can be explained by the fabrication procedure. Although the deviation of the particle distribution from uniformity was very subtle, the proposed method succeeded in detecting this pattern.

It is natural that the proposed technique can be further extended to other coordinate systems to accommodate other regular geometric spaces. For instance, the uniformity of the particles in cylindrical fuel elements can be quantitatively evaluated by using a cylindrical coordinate system. A similar technique can be formulated for the same purpose.

There are limitations of this study. First, the size of the TRISO-coated particles was ignored in the most part of the analysis. In the real SFE, the central distance between two particles cannot be closer than the diameter of the particle. This physical restriction was not considered in the simulation design. Second, we applied the proposed method on one SFE CT image only. Although there is not any particular reason that the proposed method could not work on other SFEs, it would be better that more SFE CT images can be used, and we are planning to scan more SFEs in the near future.

To conclude, the proposed technique and its extension to other coordinate systems can be used to test statistically whether a number of particles in a given regular space are uniformly distributed. Since the particle coordinates can be acquired with tomographic imaging, this technique is also non-destructive.

## Data Availability

The figures and experimental setup used to support the findings of this study are included within the article.

## References

[1] Akio K, Atsushi O, Toshiro K, Hiroyuki T (1999). Fabrication process of metal matrix composite with nano-size SiC particle produced by vortex method. J. Japanese Institute of Light Metals..

[2] Mussert KM, Vellinga WP, Bakker A, Van Der Zwaag S (2002). A nano-indentation study on the mechanical behavior of the matrix material in an AA6061-Al_2_O_3_ MMC. J. Materials Science..

[3] Wang CH, Wang SJ, Lee WD (2006). Automatic identification of spatial defect patterns for semiconductor manufacturing. International J. Production Research..

[4] Starr Fw, Douglas JF, Glotzer SC (2003). Origin of particle clustering in a simulated polymer nanocomposite and its impact on theology. J. Chemical Physics..

[5] Hashim J, Looney L, Hashmi M (2002). Particle distribution in cast metal matrix composites—Part I. J. Materials Processing Technology..

[6] Kin MK, Zeng L, Zhou Q, Tran R, Yang J (2013). On assessing spatial uniformity of particle distributions in quality control of manufacturing processes. Journal of Manufacturing Systems..

[7] Du Y, Wang XG, Xiang XC, Liu B (2014). Automatic X-ray inspection for the HTR-PM spherical fuel elements. Nuclear Engineering and Design..

[8] Yang RY, Zou RP, Yu AB (2002). Voronoi tessellation of the packing of fine uniform spheres. Physical Review E..

[9] Oger L, Gervois A, Troadec JP, Rivier N (1996). Voronoi tessellation of packing of spheres: topological correlation and statistics. Philosophical Magazine B..

[10] Fedorov A, Suboch G, Bujakov M, Fedorova L (1992). Analysis of nonuniformity in intron phase distribution. Nucleic Acids Research..

[11] Li AG, Liu ZJ, Liu Y, Xu XX, Pu YL (2012). Experimental study on microorganism ecological distribution and contamination mechanism in supply air ducts. Energy and Buildings..

[12] Bahcall JN, Wolf RA (1977). The star distribution around a massive black hole in a globular cluster. Astrophysical Journal..

[13] Huang XH, Zhou Q, Zeng L, Li XD (2017). Monitoring Spatial Uniformity if Particle Distributions in Manufacturing Processes Using the K Function. IEEE Transactions on Automation Science and Engineering..

[14] Zhu LB (2018). Uniformity Assessment of TRISO Fuel Particle Distribution in Spherical HTGR Fuel Element Using Voronoi Tessellation and Delaunay Triangulation. Science and Technology of Nuclear Installations..

[15] Rycroft CH (2009). VORO++: a three-dimensional voronoi cell library in C++. Chaos: An Interdisciplinary Journal of Nonlinear Science..

[16] Zhang ZY (2009). Current status and technical description of Chinese 2 × 250 MW_th_ HTR-PM demonstration plant. Nuclear Engineering and Design..

[17] Fütterer M (2014). Status of the very high temperature reactor system. Progress in Nuclear Energy..

[18] Yu GY (2017). 3D Nondestructive Visualization and Evaluation of TRISO Particles Distribution in HTGR Fuel Pebbles Using Cone-Beam Computed Tomography. Science and Technology of Nuclear Installations..

[19] Feldkamp LA, Davis LC, Kress JW (1984). Practical cone-beam algorithm. J. the Optical Society of America A..

[20] Otsu N (1979). A threshold selection method from gray-level histograms. IEEE Transactions on Systems, Man, and Cybernetics..

[21] Marsaglia G, Tsang WW, Wang J (2003). Evaluating Kolmogorov’s Distribution. Journal of Statistical Software..

[22] Dunn OJ (1961). Multiple Comparisons Among Means. Journal of the American Statistical Association..

